# The Action of Antidiabetic Plants of the Canadian James Bay Cree Traditional Pharmacopeia on Key Enzymes of Hepatic Glucose Homeostasis

**DOI:** 10.1155/2013/189819

**Published:** 2013-06-24

**Authors:** Abir Nachar, Diane Vallerand, Lina Musallam, Louis Lavoie, Alaa Badawi, John Arnason, Pierre S. Haddad

**Affiliations:** ^1^Natural Health Products and Metabolic Diseases Laboratory, Department of Pharmacology, Université de Montréal, P.O. Box 6128, Downtown Postal Station, Montreal, QC, Canada H3C 3J7; ^2^Canadian Intitutes of Health Research Team in Aboriginal Antidiabetic Medicines and Montreal Diabetes Research Center, Montreal, QC, Canada H3C 3J7; ^3^Office of Biotechnology, Genomics and Population Health, Public Health Agency of Canada, Toronto, ON, Canada M5V 3L7; ^4^Department of Biology, University of Ottawa, Ottawa, ON, Canada K1N 6N5

## Abstract

We determined the capacity of putative antidiabetic plants used by the Eastern James Bay Cree (Canada) to modulate key enzymes of gluconeogenesis and glycogen synthesis and key regulating kinases. Glucose-6-phosphatase (G6Pase) and glycogen synthase (GS) activities were assessed in cultured hepatocytes treated with crude extracts of seventeen plant species. Phosphorylation of AMP-dependent protein kinase (AMPK), Akt, and Glycogen synthase kinase-3 (GSK-3) were probed by Western blot. Seven of the seventeen plant extracts significantly decreased G6Pase activity, *Abies balsamea* and *Picea glauca*, exerting an effect similar to insulin. This action involved both Akt and AMPK phosphorylation. On the other hand, several plant extracts activated GS, *Larix laricina* and *A. balsamea*, far exceeding the action of insulin. We also found a significant correlation between GS stimulation and GSK-3 phosphorylation induced by plant extract treatments. In summary, three Cree plants stand out for marked effects on hepatic glucose homeostasis. *P. glauca* affects glucose production whereas *L. laricina* rather acts on glucose storage. However, *A. balsamea* has the most promising profile, simultaneously and powerfully reducing G6Pase and stimulating GS. Our studies thus confirm that the reduction of hepatic glucose production likely contributes to the therapeutic potential of several antidiabetic Cree traditional medicines.

## 1. Introduction

Diabetes is a chronic disease that occurs in two forms, namely, Type 1 and Type 2 diabetes. Type 1 diabetes affects mostly young people and is due to an autoimmune destruction of islet cells which secrete insulin [1]. Type 2 diabetes is characterized by decreased insulin sensitivity in major target organs such as liver, muscle, and adipose tissues, in addition to a decreased insulin secretion by the beta pancreatic cells [2].

These defects in patients with Type 2 diabetes cause an increase in fasting and postprandial glucose, which increases the risk of microvascular (e.g., retinopathy, neuropathy, and nephropathy) and macrovascular (mainly stroke and peripheral vascular dysfunction) complications of diabetes [3]. 

Approximately 370 million people in the world are affected by diabetes. In Canada more than 9 million people are diabetic or prediabetic. The risk to develop Type 2 diabetes in Aboriginal population is three to five times higher than the general Canadian population [4, 5]. Since 2003, our team has been examining the antidiabetic potential of selected plants used in the traditional medicine of several Cree communities in the James Bay region of Northeastern Canada. Previous studies from our group have concentrated on a group of 17 promising plant species identified through ethnobotanical surveys [6, 7]. These plants have notably been screened in bioassays of skeletal muscle and adipose tissue to identify the plants' potential to improve glycemic control [8, 9]. In continuity with these studies, the aim of this project is to evaluate the effect of these seventeen medicinal plants on hepatic glucose homeostasis. 

Glucose homeostasis is the result of a balance between glucose production by the liver (gluconeogenesis), its storage as glycogen (liver and muscle), and its uptake by peripheral tissues, notably insulin-responsive skeletal muscle and adipose tissue. Indeed, insulin, a hormone secreted by the beta pancreatic cells, works by decreasing glucose production in the liver, stimulating its uptake by skeletal muscle and peripheral tissues and enhancing its storage as glycogen [10]. Therefore, in Type 2 diabetes, beta pancreatic cell insulin deficiency is combined with insulin resistance, thus contributing to a state of hyperglycemia through an increased hepatic glucose production and a reduced peripheral glucose disposition [11]. 

Gluconeogenesis is the major metabolic pathway through which the liver produces glucose from precursors such as amino acids, lactate, glycerol, and pyruvate. This process includes several linked enzymatic reactions and is mainly activated after a fast and in diabetic patients [12]. Hepatic gluconeogenesis is controlled at three major points, namely, the reactions catalyzed by phosphoenolpyruvate carboxykinase (PEPCK), by fructose-1,6-biphosphatase, and by Glucose-6-phosphatase (G6Pase). Insulin normally reduces the activity of these enzymes to help normalize blood glucose. It does so through the activation of the signaling kinase Akt and the subsequent phosphorylation of transcription factors controlling the expression of PEPCK and G6Pase. The phosphorylated transcription factors are then expelled from the nucleus, the expression of enzymes is inhibited, and the production of glucose in the liver is eventually reduced [13, 14]. In diabetic patients, hepatic insulin resistance interferes with these events, resulting in an increased hepatic glucose, a major contributor to fasting hyperglycemia [15, 16]. G6Pase is an endoplasmic reticulum enzyme responsible of the final release of glucose into the circulation [17] and is considered to represent the rate-limiting step of gluconeogenesis [18]. Moreover, an inactivating mutation in the gene of this enzyme leads to hypoglycemia, but an increase in its expression is followed by hyperglycemia and the onset of diabetes [19]. We have thus selected a G6Pase assay to probe the Cree plants for additional antidiabetic potential targeting the liver. 

On the other hand, glycogen synthase (GS) catalyzes the rate limiting step of glycogenogenesis and is thus responsible for the storage of glucose as in both the liver and skeletal muscle. This enzyme is regulated by several transcriptional factors and kinases, the most important one being glycogen synthase kinase-3 (GSK-3). GSK-3 is a serine/threonine kinase implicated in many diseases such as diabetes, cancer, inflammation, and Alzheimer [20]. GSK-3 phosphorylates and inhibits GS thereby decreasing glycogen synthesis in liver and muscles [21]. Insulin phosphorylates and inactivates GSK-3, thus leading to the activation of the GS and the storage of glucose as glycogen [22, 23]. Therefore, we have also chosen to measure GS activity to determine the action of putative antidiabetic Cree plants on hepatic glucose homeostasis.

Aside from the hormonal regulation involving insulin, hepatic glucose homeostasis is also regulated by factors implicated in the control of energetic balance. A key kinase in this context is called AMP-activated protein kinase (AMPK), which is activated after metabolic stress (increase of the ratio AMP/ATP in the cell) [24]. Once phosphorylated, active AMPK inhibits anabolic pathways (decrease of the fatty acids synthesis, decrease of the gluconeogenesis, and increase of the storage of glucose as glycogen) and simultaneously increases catabolic pathways (increase of muscle glucose uptake, increase of the fatty acid oxidation, and increase of the glycolysis). Moreover, AMPK is activated by metformin, one of the most common oral hypoglycemic drugs used worldwide for glycemic control in diabetes and metabolic syndromes [25]. We therefore probed liver cell cultures for a potential action of Cree plants on AMPK, in relation to their effects on liver glucose metabolic enzymes.

The present study thus reports the effect of seventeen plants used in the traditional pharmacopeia of the Eastern James Bay Cree of Northern Quebec (Canada) on the activity of key enzymes of gluconeogenesis and glycogen synthesis as well as key kinases regulating these enzymes.

## 2. Material and Methods 

### 2.1. Cell Culture

The cell lines H4IIE (rat hepatoma) and HepG2 (human hepatoma) were provided by American Type Culture Collection (ATCC). H4IIE cells were grown in high glucose Dulbecco's Modified Eagle's Medium (DMEM) supplemented with 10% Fetal Bovine Serum (FBS) and 0.5% antibiotics (PS: Penicillin 100 U/mL, Streptomycin 100 *μ*g/mL). The HepG2 cells were grown in DMEM/F12 (50/50) medium supplemented with 10% FBS and 0.5% PS. The cells were incubated at 37°C, 5% CO_2_ until reaching 90% confluence. 

### 2.2. Plant Preparation

As mentioned, plant species were selected on the basis of ethnobotanical surveys previously reported [6, 7]. A taxonomist, Dr. Alain Cuerrier, identified these plants and voucher specimens have been deposited at the herbarium of the Montreal Botanical Garden. They were harvested in the Easter James Bay area in respect of Aboriginal guidelines and extracted with 80% ethanol, as previously described [8, 9]. 

### 2.3. Cytotoxicity Assay (LDH)

As was done previously for skeletal muscle and adipocyte cell lines [8, 9], maximal nontoxic concentrations of extracts were determined with the help of a cytotoxicity test, namely, that of lactate dehydrogenase (LDH) release (LDH Colorimetric kit; Roche, Mannheim, Germany). After an overnight treatment (16–18 hours) with different extracts at different concentrations, the cell culture media for each condition were collected separately (representing released LDH). The cells were then lysed with culture medium containing 1% triton X-100, for 10 minutes at 37°C, 5% CO_2_ (representing cellular LDH). All samples were collected in eppendorf tubes and centrifuged at 250 ×g at 4°C for 10 minutes. Results were expressed as the ratio of released LDH to total LDH (total LDH = released LDH + cellular LDH), normalized to values obtained from cells treated with the vehicle control (0.1% DMSO) and used to determine the optimal nontoxic concentration for each extract.

### 2.4. Hepatic Glucose Production

 Glucose-6-phosphatase activity was assessed in the H4IIE cell line. Briefly, cells (90% confluent in 12-well plates) were treated for 18 h with negative control (0.1% DMSO vehicle), positive control (Insulin, 100 nM), and seventeen plant extracts at their respective optimal nontoxic concentrations. After 18 h, cells were washed then lysed in 15 mM  phosphate buffer containing 0.05% triton X-100 and 1.3 mM  phenol (pH = 6.5). Cell lysates were incubated in glucose-6-phosphate-containing buffer (200 mM) for 40 min at 37°C where the G-6-P serves as a substrate for endogenous glucose-6-phosphatase to yield glucose. Quantification of the glucose generated in this reaction was measured using Wako AutoKit Glucose colorimetric assay (Wako Chemicals USA Inc., Richmond, VA, USA), according to manufacturers' instructions. Protein content was determined using the BCA method. Results are presented relative to vehicle control (0.1% DMSO).

### 2.5. Glycogen Synthase Activity

HepG2 cells were grown to confluency in DMEM/F12 (50/50) medium containing 10% FBS (Fetal Bovine Serum) and 0.5% PS at 37°C, 5% CO_2_. Cells were plated in 6-well plates for 4 days, then treated overnight (16–18 h) with negative control (0.1% DMSO vehicle) or each of the seventeen plant extracts at their respective optimal concentration. Positive controls were insulin (100 nM for 15 min) and 5-Aminoimdazole-4-carboxamide-1-*β*-D-ribofuranoside (AICAR, 2 mM for 120 min). After treatment, cells were suspended in buffer solution (50 mM glycylglycine, 100 mM sodium floride, 20 mM EDTA, 0.5% glycogen, pH 7.4 + complete protease inhibitor cocktail). The lysates were centrifuged at 1000 ×g for 20 minutes at 4°C. 30 *μ*L of supernatant was added to 100 *μ*L of buffer solution for active GS (25 mM glycylglycine, 0.275 mM UDP-glucose, 0.12 *μ*Ci/mL U-^14^C UDP-glucose, 1% glycogen, 1 mM EDTA, 10 mM sodium sulfate, pH 7.5) and another 30 *μ*L of supernatant was added to 100 *μ*L of buffer solution for total GS (25 mM Tris, 5 mM UDP-glucose, 0.12 *μ*Ci/mL U-^14^C UDP-glucose, 1% glycogen, 3 mM EDTA, 5 mM glucose-6-phosphate, pH 7.9). All the tubes were incubated at 30°C for 120 minutes. After incubation 90 *μ*L of the mix was transferred on Watman paper (Watman 31 ET chr 2 cm^2^). The papers were rinsed with cold ethanol (4°C) for 30 minutes then 2 times with ethanol 66% at room temperature for 30 minutes. The papers were covered with acetone for 2-3 minutes then transferred into scintillation vials. The resulted radioactivity was counted with a specific protocol for ^14^C using a *β*-counter (LKB Wallac 1219; Perkin-Elmer, Woodbridge, ON, Canada).

### 2.6. Western Blot

Cells were lysed in RIPA buffer (RadioImmun Precipitation Assay) (0.1 M Hepes, 0.3 M NaCl, 10 mM EGTA, 4 mM MgCl_2_·6H_2_O, 10% glycerol, 2% Triton X-100, 0.2% SDS, 2 mM PMSF, 10 mM NaF, 100 *μ*M Na-orthovanadate, 1 mM Na-pyrophosphate). After centrifugation at 12000 ×g, 4°C, for 12 minutes, the supernatants were collected and used for western blot analysis. 40 *μ*g of proteins was loaded onto an electrophoresis gel then transferred on nitrocellulose membrane (Millipore, Bedford, MA, USA). The membranes were blocked for 2 hours in TBST (20 mM Tris, pH 7.6, 137 mM NaCl, 0.1% Tween-20) + 5% milk. Antibodies used were the following: p-GSK-3 (Ser 9), GSK-3 (1 : 1000, 5% milk, Millipore, Bedford, MA, USA) and secondary antibody : anti-rabbit (1 : 50000, 5% milk, Jackson ImmunoResearch Laboratories, West Grove, PA, USA); p-AMPK*α* (Thr 172) (1 : 350, 5% BSA, Cell Signaling Technology, Danvers, MA, USA), AMPK (1 : 500, 5% BSA, Cell Signaling Technology, Danvers, MA, USA) and secondary antibody : anti-rabbit (1 : 4000, 5% BSA, Jackson ImmunoResearch Laboratories, West Grove, PA, USA); p-Akt (Thr 308) (1 : 1000, 5% BSA, Cell signaling Technology, Danvers, MA, USA), Akt (1 : 1000, 5% BSA, Cell Signaling Technology Danvers, MA, USA) and secondary antibody : anti-rabbit (1 : 10000, 5% BSA, Jackson ImmunoResearch Laboratories, West Grove, PA, USA). 

The membranes were incubated with primary antibodies overnight at 4°C then with secondary antibodies 1 hour at room temperature. Finally the bands were detected with the ECL Plus western blot detection system (Perkin Elmer, Woodbridge, Canada).

### 2.7. Statistical Analysis

All data are reported as the mean ± SEM of 3 different experiments with triplicate for each sample. Results were analyzed by one-way analysis of variance (ANOVA) using StatView software (SAS Institute Inc., Cary, NC, USA). A *P* value below 0.05 was considered statistically significant.

## 3. Results

### 3.1. LDH Test (Cytotoxicity)

Hepatic cells (H4IIE and HepG2) were treated overnight (16–18 h) with plant extracts at different concentrations and LDH release measured.  Table 1 presents the maximal nontoxic concentrations determined for each plant extract. At the selected concentrations, none of the extracts induced more than 9% release of LDH. These concentrations were subsequently used for all experiments.

### 3.2. Seven of the Seventeen Plant Extracts Have a Potential to Decrease Hepatic Glucose Production

**Figure 1:**
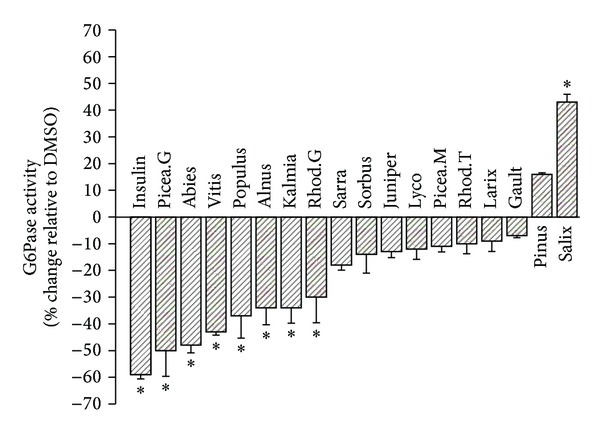
Effect of 17 plant extracts on the activity of G6Pase. Results shown represent the change in G6Pase activity observed after overnight treatment of H4IIE cells with optimal nontoxic concentrations of indicated plant extracts. They are expressed relative to DMSO (0.1%) vehicle controls (0% inhibition). Assays were carried out in triplicate on three different cell cultures. Insulin (100 nM) was used as a positive control. *P < 0.05 significantly different from DMSO vehicle control.

We used the inhibition of rate-limiting glucose-6-phosphatase activity in H4IIE hepatocytes as a measure of the antidiabetic potential of Cree plant extracts at the level of the liver. As expected, insulin (positive control) decreased G6Pase activity by approximately 60%. Out of the seventeen plant extracts tested, eight had a statistically significant effect on G6Pase activity when compared to respective DMSO vehicle controls run in parallel (Figure 1). Seven of the eight plants reduced activity by 50 to 30% on average, whereas *Salix planifolia* increased activity by 43%. The inhibitory activity of extracts of *Picea glauca* and *Abies balsamea* was almost as important as the insulin positive control (reductions of 50% and 48%, respectively, compared to DMSO).

### 3.3. Absence of Additive Effect of either *P. glauca* or *A. balsamea* Extracts with Insulin

**Figure 2:**
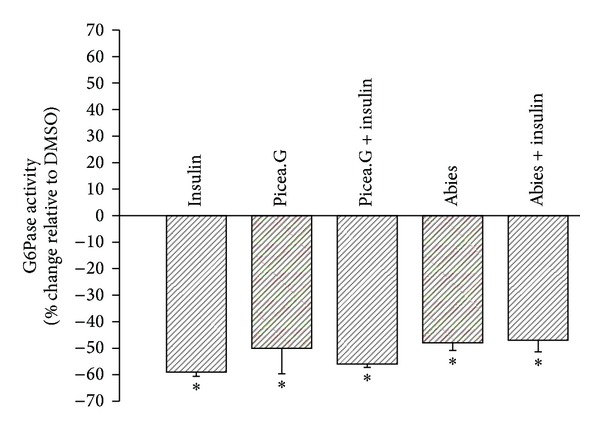
Effect of insulin and two plant extracts on G6Pase activity. Results shown represent the change in G6Pase activity observed after overnight treatment of H4IIE cells with *Picea glauca* or *Abies balsamea* plant extracts in the presence or absence of insulin (100 nM). They are expressed relative to DMSO (0.1%) vehicle controls (0% inhibition). Assays were carried out in triplicate on three different cell cultures. *P < 0.05 significantly different from DMSO vehicle control.

Since the effects of *P. glauca* and *A. balsamea* were in the same order of magnitude as the insulin control, we sought to examine whether the plant extracts and the hormone showed any interaction in biological activity.  Figure 2 presents the results of experiments where each plant extract was administered individually with or without insulin. As can be easily appreciated, insulin did not influence the effect of each plant extract and vice versa. 

### 3.4. Differential Modulation of Insulin-Dependent and -Independent Signaling by Cree Plant Extracts in H4IIE Hepatocytes

**Figure 3:**
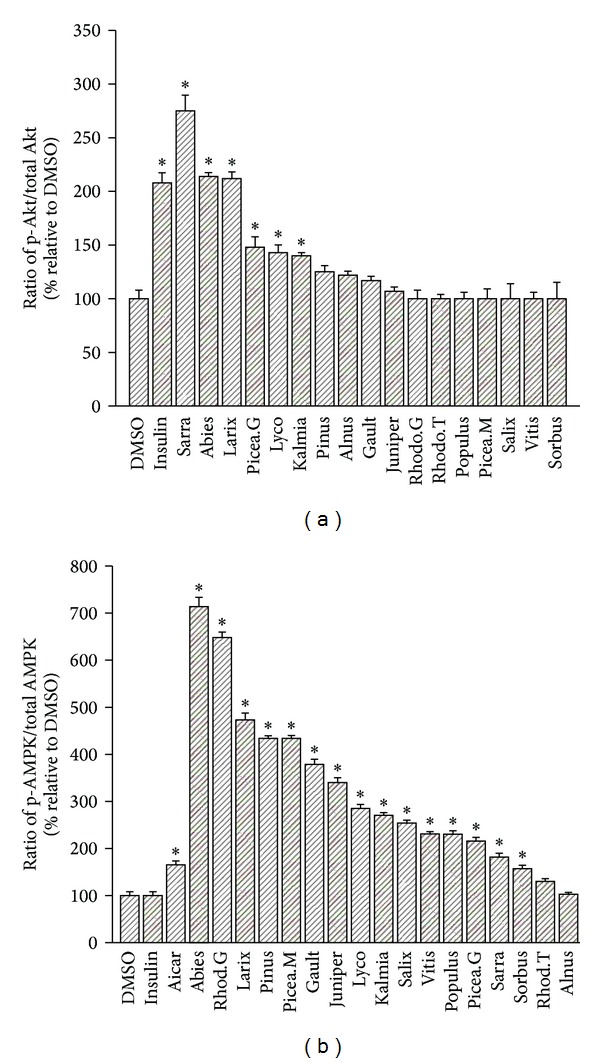
Effect of 17 plant extracts on kinases regulating G6Pase activity. (a) Phosphorylation of Akt. Phosphorylated (p-Akt) and total Akt were measured by Western blot in H4IIE cells treated with optimal nontoxic concentrations of indicated plant extracts. The ratio of p-Akt/total Akt is expressed as a percentage relative to values obtained for DMSO (0.1%) vehicle controls. Insulin (100 nM) was used as a positive control. *P < 0.05 significantly different from DMSO vehicle control. (b) Phosphorylation of AMPK. Phosphorylated (p-AMPK) and total AMPK were measured by Western blot in H4IIE cells treated with optimal nontoxic concentrations of indicated plant extracts. The ratio of p-AMPK/total AMPK is expressed as a percentage relative to values obtained for DMSO (0.1%) vehicle controls. AICAR (2 mM) was used as a positive control. *P < 0.05 significantly different from DMSO vehicle control.

We next sought to determine if insulin-dependent and/or -independent signaling pathways were involved in the observed effects of plant extracts on G6Pase activity. H4IIE hepatocytes were thus incubated as above with each of the seventeen plant extracts and probed for phosphorylated Akt (key signaling kinase of the insulin-dependent metabolic pathway) and for phosphorylated AMPK (key metabolic switch kinase responsible for insulin-independent metabolic pathway). As shown in Figure 3(a), six of the seventeen plants significantly enhanced the phosphorylation of Akt. Two of these six, namely, *A. balsamea* and *P. glauca*, were plant extracts also observed to significantly decrease G6Pase activity in H4IIE hepatocytes. However, we found no correlation between Akt phosphorylation and modulation of G6Pase activity (data not illustrated).

Unlike Akt, all but two of the seventeen plant extracts tested were found to induce a significant increase in the phosphorylation of AMPK when compared to vehicle control (Figure 3(b)). A number of plant extracts, notably *A. balsamea*, *Rhododendron groenlandicum*, *Larix laricina*, *Pinus banksiana*, *Picea mariana,* and *Gaultheria hispidula* exerted effects that were 2-4-fold greater than the positive control AICAR. However, as with Akt, no correlation was found between the ability of a plant's extract to activate AMPK and to inhibit G6Pase (data not illustrated).

### 3.5. Two Cree Plants Stand out for Very Potent Stimulation of Glycogen Synthase Activity

**Figure 4:**
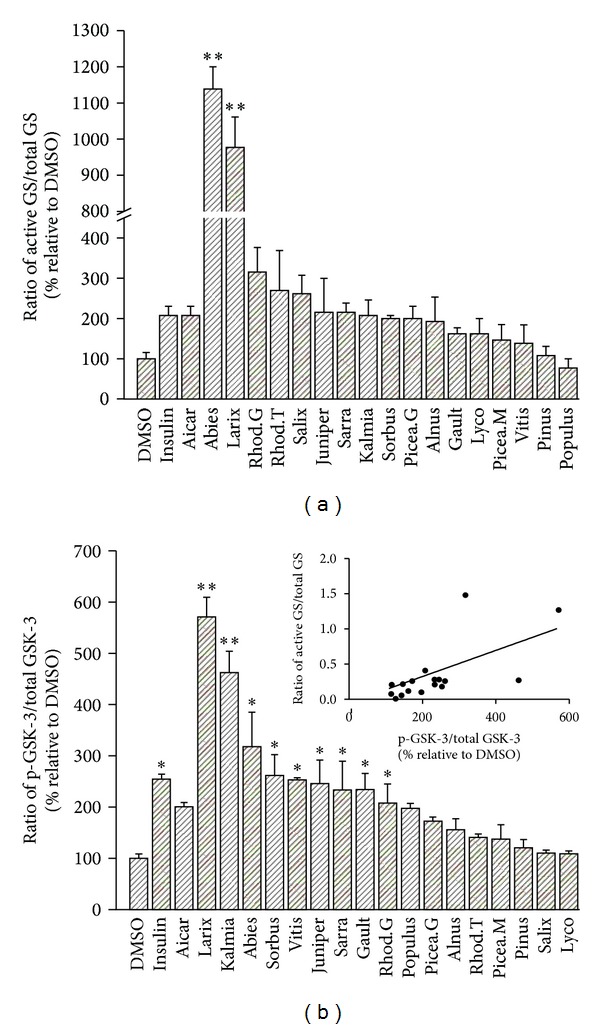
Effect of 17 plant extracts on components involved in hepatic glucose storage. (a) Activation of GS. Results shown represent the glycogen synthase (GS) activity observed after overnight treatment of HepG2 cells with optimal nontoxic concentrations of indicated plant extracts. They are expressed relative to DMSO (0.1%) vehicle controls (100% activity). Assays were carried out in triplicate on three different cell cultures. Insulin (100 nM) and AICAR (2 mM) were used as positive controls. **P < 0.01 significantly different from DMSO vehicle control. (b) Phosphorylation of GSK-3. Phosphorylated (p-GSK-3) and total GSK-3 were measured by Western blot in HepG2 cells treated with optimal nontoxic concentrations of indicated plant extracts. The ratio of p-GSK-3/total GSK-3 is expressed as a percentage relative to values obtained for DMSO (0.1%) vehicle controls. Insulin (100 nM) and AICAR (2 mM) were used as positive controls. *P < 0.05 and **P < 0.01 significantly different from DMSO vehicle control. Inset: Correlation between GSK-3 phosphorylation and activation of GS induced by the 17 plant extracts (*r* ^2^ = 0.36, *P* < 0.05).

Glycogen synthase (GS) is the second rate-limiting enzyme involved in hepatic glucose homeostasis that we used to probe for the antidiabetic potential of Cree plant extracts. As expected, the hormone insulin, used as a positive control activated GS by 2-fold when compared to the DMSO vehicle control in HepG2 cells (Figure 4(a)). AICAR, an activator of AMPK, also induced a similar 2-fold increase in GS activity. Several plants also significantly stimulated GS activity as much or greater than the insulin and AICAR controls (between 3- and 2-fold). However, two plants clearly stood out of the lot. Indeed, *A. balsamea* and *L. laricina* activated the enzyme by 11- and 9-folds, respectively, compared to the DMSO vehicle control (Figure 4(a)).

### 3.6. Implication of GSK-3 in the Action of Cree Plants on Glycogen Synthase

As expected, and compared to the DMSO vehicle control, insulin treatment lead to a significant (over 2-fold) increase in the phosphorylation of GSK-3, which is known to inactivate the kinase, hence releasing its inhibitory action on GS and favoring glucose storage as glycogen. Nine of the seventeen plant extracts tested also significantly increased GSK-3 phosphorylation (Figure 4(b)). Consistent with the results of the GS activity assay, *L. laricina* and *A. balsamea* were among the most active species to enhance GSK-3 phosphorylation. In fact, when the ability of a given plant extract to activate GS was compared to the same plant's capacity to induce GSK-3 phosphorylation, a modest yet significant positive correlation was obtained (*r* ^2^ = 0.36, *P* < 0.05, Figure 4(b) inset).

## 4. Discussion

As mentioned in the introduction, Canadian Aboriginals suffer from a higher incidence of Type 2 diabetes [4, 5] as well as diabetic complications, a situation that has been linked to poor treatment adherence due, in part, to the cultural inappropriateness of modern drug based approaches [26, 27]. To provide culturally adapted complementary and alternative treatment options for these populations, we have put together a comprehensive platform of *in vitro* (mostly cell based) bioassays and *in vivo* animal models to rigorously assess the action of Boreal forest plants on tissues/organs involved in metabolic control [28]. Plant species were first identified through a novel ethnobotanical survey methods based on a set of clinically relevant diabetes symptoms [6]. A set of seventeen plants with promising antidiabetic potential was initially screened in pancreatic, skeletal muscle, adipocyte and, preneuronal cell lines [8, 9]. We notably established that some species exhibited the potential to accelerate adipogenesis and to stimulate glucose uptake in muscle cells and adipocytes [8, 9]. 

Along with peripheral glucose disposition, hepatic glucose production plays a major role in the pathogenesis of the obesity-diabetes metabolic disease continuum [19]. We thus sought to further assess the antidiabetic potential of the Cree plants in liver cell lines. Two key enzymes involved in hepatic glucose storage and release were selected. We also examined major metabolic control/signaling kinases that modulate these key enzymes. 

Firstly, we selected G6Pase, which is a key enzyme implicated in hepatic gluconeogenesis, being the rate-limiting step for glucose release from hepatocytes [18]. The expression of this enzyme is enhanced by several transcription factors such as forkhead transcription factor O1 (FoxO1), hepatic nuclear factor 4 (HNF4), and peroxisome proliferator-activated receptor-*γ* coactivator-1*α* (PGC-1*α*) [29, 30]. Through its receptor signaling pathways, insulin controls the expression of G6Pase. It does so by inhibiting the aforementioned transcriptional factors as well as their binding to the promoter of the G6Pase gene and to that of the PEPCK gene, another key enzyme involved in the gluconeogenesis pathway [31]. The signaling kinase Akt plays a crucial role in insulin's metabolic action in the liver and was thus also assessed in H4IIE hepatocytes to gain insights on the potential of Cree plants to modulate insulin-dependent pathways. On the other hand, AMPK is an insulin-independent serine/threonine kinase implicated in energy balance [24]. It is involved in hepatic metabolic homeostasis [32], which includes the ability to decrease G6Pase activity through the phosphorylation of CREB (c-AMP-regulator element-binding protein) [33]. We thus also probed H4IIE hepatocytes for AMPK activation after treatment with plant extracts in order to assess insulin-independent pathways.

As expected, we found that an optimal supraphysiological concentration of insulin (100 nM) reduced G6Pase activity in H4IIE hepatocytes to approximately half of that seen in DMSO vehicle controls. Interestingly, a number of the Cree plant species also exhibited the capacity to lower G6Pase activity. Most prominent were *P. glauca* and *A. balsamea*, whose action was equivalent to that of the insulin positive control. When the extracts of these two plants were combined with insulin, no additive effect was observed. One plausible interpretation of this result is that *P. glauca* and *A. balsamea* may act through pathways similar to insulin, these being already maximally stimulated by the supraphysiological dose of the hormone. This was confirmed for *A. balsamea*, whose extract stimulated the phosphorylation of Akt as prominently as insulin did. This was also true for *P. glauca*, albeit to a lesser extent. In fact, no correlation could be found between the plant extracts effects on G6Pase inhibition and Akt phosphorylation. In skeletal muscle cells, several Cree plants had no effect on Akt [34] suggesting that plant extracts may have differential effects on insulin signaling pathways in different tissues. *A. balsamea* extract also strongly stimulated the insulin-independent AMPK pathway. In fact, its action was over threefold greater than the AICAR positive control. In contrast, *P. glauca* stimulated the AMPK pathway to a slightly greater extent than AICAR, yet decreased G6Pase activity to the same extent as *A. balsamea*. These observations combined with the clear lack of correlation between either Akt or AMPK activation, and G6Pase inhibition highlights the fact that other pathways modulating G6Pase activity may be influenced by the Cree plants. Future studies will have to take this into consideration. 

On the other hand, HepG2 cells were selected to study the potential action of Cree plants on the second key enzyme of hepatic glucose homeostasis, namely, glycogen synthase, because they exhibit a better expression of this enzyme [35]. Insulin increases the activity of glycogen synthase by inhibiting the GSK-3 (phosphorylation mechanism) thus increasing the glucose storage as glycogen [36, 37]. Again, we observed that several Cree Boreal forest plants exerted a positive action on glycogen synthase, a majority of which acting at least as much as the insulin positive control, which doubled GS activity over baseline (DMSO control). However, extracts of *A. balsamea* and* L. laricina* enhanced GS activity 9–11-fold over baseline (corresponding to 4-5-fold above insulin). This skewed the statistical analysis such that only these two plants appeared to have a statistically significant impact on GS activity.

We also assessed the impact of Cree plant extracts on GSK-3 phosphorylation (leading to its inactivation [21]) since this kinase plays a central role in the control of GS activity [22]. Unlike G6Pase, we found that the phosphorylation of GSK-3 induced by respective Cree plant extracts could explain approximately 40% (*r* ^2^ = 0.36) of their capacity to increase GS activity. This is a respectable proportion that indicates that GSK-3 might be a common target of several Boreal forest plants. However, such a result also implies that other signaling/metabolic control pathways may also be involved, and additional studies will be necessary to identify these.

Our study thus clearly supports the notion that part of the therapeutic potential of several putative antidiabetic Boreal forest plants can involve the reduction of hepatic glucose output. The results more specifically highlight the favorable profile of three plants. Firstly, *P. glauca* also stood out as the most potent Cree plant capable of reducing G6Pase activity as much as a supraphysiological concentration of insulin. This action is clearly associated with the phosphorylation of the key kinases Akt and AMPK in H4IIE hepatocytes. Previous *in vitro* studies from our group demonstrated that *P. glauca* crude extract did not affect muscle glucose uptake or adipogenesis [9], yet exhibited potent effects for diabetes complications (neuroprotective action) [38]. The present results add an additional therapeutic potential by demonstrating the ability of *P. glauca* to reduce the activity of the enzyme limiting hepatic glucose output.

Secondly, *L. laricina* extract was found to be a potent modulator of the glycogen storage pathway. Indeed, it increased GS activity severalfold more than a supraphysiological concentration of our insulin reference positive control. Likewise, the plant's capacity to enhance GSK-3 phosphorylation was the greatest of all Cree plants and, again, more than twofold that of insulin. Moreover, although *L. laricina* did not significantly alter G6Pase activity in H4IIE hepatocytes, it was among the top Cree plants stimulating the phosphorylation of Akt and AMPK, two important kinases involved in hepatic metabolic control [14, 25]. Such an apparently important antidiabetic potential is consistent with recent* in vitro* and *in vivo* studies from our group with the same crude extract of *L. laricina* used in the present work. Indeed, *in vitro*, it stimulated glucose uptake [8] and AMPK [34] in C2C12 myocytes while enhancing adipogenesis in 3T3-L1 cells [8]. In the mouse diet-induced obesity model, *L. laricina* demonstrated a clear capacity to reduce glycemia and improve insulin resistance [39]. The current study thus further validates the antidiabetic potential of *L. laricina* crude extracts and adds the liver as a putative target organ for the plant.

Finally, the results presented herein highlight the very strong action of *A. balsamea* on key enzymes and modulating kinases controlling hepatic glucose production. Indeed, the plant's crude extract reduced G6Pase and increased Akt phosphorylation to the same extent as insulin in H4IIE hepatocytes. It was also the most potent of all Cree plant extracts to enhance AMPK phosphorylation in the same cell line. Likewise, *A. balsamea* was the most potent activator of glycogen synthase in HepG2 cells when compared to other Boreal forest plant extracts, exerting an effect severalfold higher than insulin. This action was also associated with an enhanced phosphorylation of the key kinase GSK-3. Hence, *A. balsamea* crude extract was the only Cree plant to simultaneously and potently affect the two major pathways involved in hepatic glucose homeostasis. Together with the prior *in vitro* demonstration of the ability of this plant to enhance glucose uptake in muscle and adipose cells [8], this encourages more detailed study of the antidiabetic potential of the plant, notably through the determination of active principles and validation using *in vivo* animal models. 

## 5. Conclusion

In summary, this study confirmed that Boreal forest plants of the Cree traditional pharmacopeia have the potential to reduce hepatic glucose output and hence provide beneficial therapeutic action in the context of the obesity-diabetes continuum of metabolic diseases. Our results also lend further support to the soundness of considering Cree traditional medicine and its associated medicinal plants as worthy complementary and alternative approaches to improve diabetes care and management, doing so in a culturally mindful and respectful manner.

## Figures and Tables

**Table 1:** Optimal concentrations of plant extracts used to treat H4IIE and HepG2 cells were determined by the LDH cytotoxicity.

Plant extracts	Abbreviation used	Concentrations in *μ*g/mL
*Abies balsamea *	Abies	50
*Kalmia augustifolia *	Kalmia	50
*Larix laricina *	Larix	25
*Sarracenia purpurea *	Sarra	25
*Sorbus decora *	Sorbus	15
*Juniperus communis *	Juniper	3.75
*Rhododendron tomentosum *	Rhod.T	50
*Rhododendron groenlandicum *	Rhod.G	50
*Gaultheria hispidula *	Gault	25
*Picea mariana *	Picea.M	10
*Picea glauca *	Picea.G	125
*Salix planifolia *	Salix	15
*Alnus incana *	Alnus	50
*Populus balsamifera *	Populus	100
*Lycopodium clavatum *	Lyco	100
*Pinus banksiana *	Pinus	10
*Vaccinium vitis *	Vitis	200
